# Efficacy of tibolone and raloxifene for the maintenance of skeletal muscle strength, bone mineral density, balance, body composition, cognitive function, mood/depression, anxiety and quality of life/well-being in late postmenopausal women ≥ 70 years: Study design of a randomized, double-blind, double-dummy, placebo-controlled, single-center trial

**DOI:** 10.1186/1745-6215-9-32

**Published:** 2008-06-05

**Authors:** Didy E Jacobsen, Monique M Samson, Yvonne T van der Schouw, Diederick E Grobbee, Harald JJ Verhaar

**Affiliations:** 1 Department of Geriatric Medicine, Mobility Laboratory, University Medical Center Utrecht, Utrecht, The Netherlands; 2 Julius Center for Health Sciences and Primary Care, University Medical Center Utrecht, Utrecht, The Netherlands

## Abstract

**Background:**

Postmenopausal women are prone to develop functional disabilities as a result of reduction in muscle strength and muscle mass caused by diminished levels of female sex hormones. While hormone replacement therapy may counteract these changes, conventional hormone replacement therapy is associated with potential harmful effects, such as an increased risk of breast cancer, and its prescription is not recommended. For this reason newer alternative drugs, such as tibolone, a synthetic steroid with estrogenic, progestogenic and androgenic activity, and raloxifene, a selective estrogen receptor modulator, may be more appropriate. This trial investigates the effect of tibolone and raloxifene on muscle strength.

**Methods:**

We recruited 318 elderly women in our single-center randomized, double-blind, double-dummy, placebo-controlled trial. Participants were randomized to tibolone 1.25 mg (Org OD 14, Organon NV, the Netherlands) plus placebo, raloxifene 60 mg (Evista^®^, Eli Lilly, United States) plus placebo or two placebo tablets daily for 24 months.

The primary aim is to determine if there is a difference between tibolone and placebo or if there is a difference between raloxifene and placebo. Primary endpoints are muscle strength and bone mineral density. The secondary endpoints are postural balance, body composition, cognitive function, anxiety, mood and quality of life. The secondary aim is to determine if there is a difference between tibolone and raloxifene.

The measure of effect is the change from the baseline visit to the visits after 3 months, 6 months, 12 months, and 24 months. A follow-up measurement is planned at 30 months to determine whether any effects are sustained after cessation of the study. By December 2007 the blind will be broken and the data analyzed.

**Trial registration number:**

NTR: 1232

## Background

Aging is associated with muscle atrophy, physical frailty, and impaired cognitive function. This functional decline can severely affect quality of life and reduce the likelihood of a person being able to live independently. Women are more vulnerable to the effects of this age-related muscle loss because their peak muscle mass is lower than that of men and around the time of menopause they experience an additional 15% loss of muscle mass [1-3]. Hormonal replacement therapy (HRT) in postmenopausal women may prevent muscle strength decline [2,4-9]. Unfortunately, however conventional hormone replacement therapy has serious side-effects such as an increased risk of breast cancer, thrombo-embolism, cholecystitis, stroke and coronary events, especially in elderly women. Unopposed estrogen replacement therapy increases the risk of endometrial carcinoma. [10,11]

For this reason, newer drugs, such as tibolone, a synthetic steroid with oestrogenic, progestogenic, and androgenic activity, and raloxifene, a selective estrogen receptor modulator, may be more attractive.

Tibolone is a tissue-specific compound that has favorable effects on bone, vagina, climacteric symptoms, mood, and sexual well-being in postmenopausal women, without having an estrogen-like stimulating effect on the endometrium or breast [12]. In a randomized, placebo-controlled and double-blind trial involving early postmenopausal women, handgrip strength increased by about 4% after 1 year of treatment with tibolone 2.5 mg [13]. Tibolone 1.25 mg may be effective in preventing osteoporosis and post-menopausal symptoms.

Selective estrogen receptor modulators (SERMs) are a diverse group of compounds that bind with specificity and high affinity to estrogen receptors (ER) consisting of the ER α and the ER β. SERMs can act as either ER agonists or antagonists, depending on the tissue type. Raloxifene hydrochloride is a nonsteroidal benzothiophene with estrogen agonistic effects on bone, serum lipids, and the arterial vasculature and with estrogen antagonistic effects in the breast and uterus [14]. Raloxifene has been approved for osteoporosis prevention and the treatment of postmenopausal osteoporosis in several countries. Its actions on bone are similar to those of estrogens [15].

Relatively little is known about the effects of estrogen supplementation, with regard to frailty, initiated later in the postmenopausal period (> 70 years) and even less is known about the effects of tibolone and raloxifene on muscle strength, body composition, bone mineral density (BMD), balance, cognitive function and mood/depression in late postmenopausal women. The purpose of this study is to determine whether these newer drugs have beneficial effects in older postmenopausal women.

## Design and Methods

The study is a single-center, randomized, double-blind, double-dummy, placebo-controlled trial to evaluate the effect of tibolone and raloxifene on skeletal muscle strength, body composition, BMD, balance, cognitive function, mood/depression, anxiety and quality of life in late postmenopausal women aged ≥ 70 years. The aim was to recruit 325 study subjects, so that, after allowing for a drop out rate of 30 % over 2 years, data could be collected from 225 women.

Participants were randomly assigned by the hospital pharmacy of the University Medical Center of Utrecht using computerprogramm "Design" to one of the three treatment groups in a 1:1:1 ratio after baseline measurements had been taken. The sub-blocks contained 12 numbers. The design is double-blind. We do not expect that unblinding by symptoms will occur. In a diary we actively inquired about leg cramps and flushing. These have been reported to be side effects of raloxifene, but only in 0–10% for legcramps and more than 10% for flushing [16]. Legcramps also occur often in women of this age.

The treatment period was 24 months, with 1 month being considered to be 28 days. Assessments were performed at baseline and after 3, 6, 12, and 24 months. Participants will also be assessed at 30 months, to determine whether treatment has a lasting effect. The Institutional Review Board of the University of Medical Center Utrecht approved the study protocol. All participants gave written informed consent at the screening visit.

### Inclusion criteria

-Healthy postmenopausal women;

-Minimum age 70 years at the time of inclusion;

-Body mass index between 18 and 35 kg/m^2^,

-Subjects should be willing and able to comply with the protocol for the duration of the study, after written informed consent.

### Exclusion criteria

-Steroid therapy or other drugs affecting muscle mass taken during the last 6 months;

-History or presence of any malignancy (except non-melanoma skin cancers);

-Undiagnosed abnormal vaginal bleeding in the past year prior to screening;

-Presence or history of endometrial hyperplasia with or without atypia;

-Presence or history of cardiovascular, cerebrovascular or thrombo-embolic disorders;

-Current liver or renal disease or history of this condition;

-Uncontrolled hypertension (systolic blood pressure ≥ 170 mm Hg systolic and/or diastolic blood pressure ≥ 105 mm Hg);

-Osteoporosis: Z-score < -2

-Bone disease other than osteoporosis such as Paget's disease;

-Osteomalacia or bone metastases;

-Alcohol abuse (average intake of more than 4 alcohol containing units per day);

-Smoking more than 10 cigarettes/day;

-Use of sex hormones, corticosteroids, insulin, anti-coagulants or enzyme-inducing drugs;

-Treatment with tibolone or raloxifene within the last 6 months;

-Known hypersensitivity to tibolone or raloxifene;

-Presence of any condition, concomitant disease, intercurrent illness or resultant therapy that would interfere either with the participants' compliance or with the results of the study and/or their evaluation;

-Major problems with locomotion and cognitive impairment (MMSE < 24);

-Participation in another study.

### Recruitment

Recruitment started on 23 July 2003 by means of an advertisement placed in the local newspaper (reaching 194.000 households, only a few involving women 70 years or older). Interest in the study was expressed by 80 women; 38 women were randomized. Further recruitment was done by mailing 16.090 women aged 70–80 years whose addresses were selected by the municipal register of the cities Utrecht, Zeist, Houten, Bilthoven, and Driebergen. Women who previously participated in research at the geriatric department were also recruited (table 1).

**Table 1:** Recruitment, inclusion and exclusion.

**Recruitment**	**Total**	**Positive response**	**Exclusion after positive reaction**	**Exclusion after screening**	**Inclusion**	**Never started**
Local newspaper:	unknown	80	38	4	38	6
UMC group	172	32	8	8	16	3
Local municipal registers	16090	541	249	28	264	26

total	16262	653	295	40	318	35

Women were asked to complete a questionnaire about the type of medication used, number of falls, troubles walking, use of a walking aid, dyspnea, history of thrombosis, liver complaints, hysterectomy or uterine complaints, weight, and height. Women who did not meet the inclusion criteria were sent a letter thanking them for their interest. Those who met the inclusion criteria and who were still interested in participation were invited, by telephone, to a screening visit. During this telephone conversation, the participant's medical history was checked for exclusion criteria.

**Figure 1:**
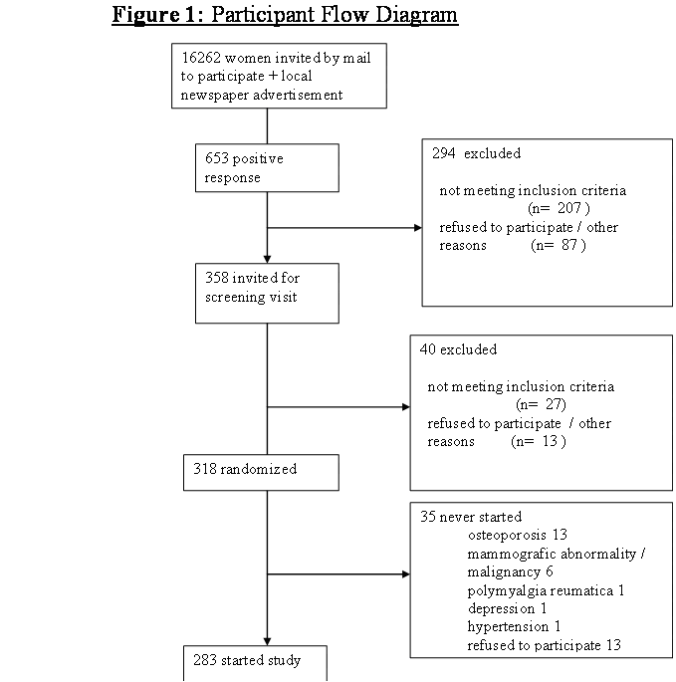
**Flowchart representing recruitment after a newspaper advertisement and after invitation of women ≥ 70 years selected by municipal registers of several cities and women who participated before in a study of the geriatric department. **Suitable women were invited for a screening visit. They were included in the trial if they met the inclusion criteria and after written informed consent. Then randomization took place.

Two hundred and seven women were excluded and 87 women refused to participate or there were other reasons for exclusion. Reasons for exclusion after a positive reaction and after screening are given in table 2 and 3. A flow chart of study recruitment is given in figure 1.

**Table 2:** Reasons for exclusion after positive response

**Exclusion reason:**	**Total:**
Subject does not want to	79
Already involved in another study	2
BMI > 33 or < 18	28
Cardiovascular disease	42
Hypertension	3
Thrombo-embolic disease	33
Malignancy	26
COPD	1
Liver problem	2
Decreased functionalability	3
On hormone replacement therapy	22
Contra-indicated medication	46
Parkinsonism	1
Osteoporosis	1
Two-sided hip prothesis	5
Moved to Spain/Belgium	2
General practitioner advised not to participate	1

Total	294

**Table 3:** Exclusion after screening.

**Exclusion reason:**	**Number:**
Malignancy	3
Benign abnormality	4
Medication	4
Hypertension	4
BMI > 35	2
Angina pectoris	4
TIA	1
Thrombosis	2
Untreated diabetes mellitus	1
Surgery	1
Own reasons	9
MMSE < 24	3
Skin problems	1
Pain/functional disability	1

total	40

### Information and screening

At the screening visit women were informed about the trial and gave their written informed consent. A medical and gynecological history was obtained and medication use was recorded. Blood pressure, heart rate, weight, and height were measured and mamma palpation, cardial, pulmonal and abdominal examinations were performed. In- and exclusion criteria were checked again using a list of questions. Finally, a Mini Mental State Examination was performed. Forty women were excluded after screening (table 3). Within 28 days of the screening visit, baseline measurements were taken and the participants were randomized.

### Randomization visit and baseline assessments

Baseline measurements were taken within 28 days of screening at the time of randomization. Mammography was performed in all women except those who had had a mammography within 12 months before randomization. If there was an abnormality, malignancy or dubious malignancy, the participant was excluded. Dual Energy X-ray Absorptiometry (DEXA) was performed to measure BMD. If the z-score was lower than -2, the subject was excluded; if the z-score was between -1 and -2, the subject was given calcium supplements and entered the trial.

At baseline muscle strength and power were measured and functional mobility was assessed with the timed "up and go test". Postural balance was determined with body sway and functional reach. Women completed the Women's Health Questionnaire (WHQ) to determine quality of life. Other questionnaires administered were on habitual physical activity (HPA), the State Trait Anxiety Inventory (STAI) and the Geriatric Depression Scale. Cognitive function was determined with a 15 word test and a trail B test. The tests used at successive visits are given in table 4.

**Table 4:** Examinations and assessments.

Assessment	Visit 1	Visit 2	Visit 3	Visit 4	Visit 5	Visit 6	Visit 7
	screen	Base	mnth3	mnth6	mnth12	mnth24	mnth30
Medical history	X						
Physical examination	X						
Gynaecologicol examination	X						
Breast examination	X						
Mammography*	X						
In/exclusion criteria	X						
Vital signs	X	X	X	X	X	X	X
Bone mineral density		X				X	
Body composition		X	X	X	X	X	X
Muscle strength		X	X	X	X	X	X
Cognitive function		X	X	X	X	X	X
Functional mobility		X	X	X	X	X	X
Mood/depression		X	X	X	X	X	X
Quality of life		X	X	X	X	X	X
HPA		X	X	X	X	X	X
Adverse events			X	X	X	X	
Drug accountability			X	X	X	X	

* Only if not performed in the year before.

Randomization started at July 15^th ^2003. Three hundred and eighteen women were randomized. Thirty-five women were excluded after randomization because of osteoporosis (n = 13), mamma carcinoma or abnormality on mammography (n = 6), hypertension (n = 1), polymyalgia rheumatica (n = 1) and some women decided to withdraw from the study at this stage (n = 14). Participants were randomized to receive tibolone 1.25 mg (Org OD 14, Organon NV, the Netherlands) plus placebo, raloxifene 60 mg (Evista^®^, Eli Lilly, United States) plus placebo or two placebo tablets daily for 24 months. Because the results of the Long term Intervention on Fractures with Tibolone (LIFT)-study suggested there was a higher risk of cerebrovascular accidents with tibolone use after an average of 2.4 years (hazard ratio 2.3), our research group decided to stop the tibolone arm of the study on February 2006 [17]. One hundred and five women were randomized to use tibolone. Of those women 15 never started the study, 27 already dropped out 15 after baseline, 5 after 3 months, 3 after six months, 4 after one year measurements), and 19 women had to stop the study before one year measurements were reached. Available one-year data about the tibolone group (n = 44) will be analysed.

### Measurements

The following primary parameters were used to determine the effect of the study medication

#### Muscle strength and muscle power

Maximum voluntary isometric knee extension strength (both legs) was measured as the force applied at the ankle, with the subject seated in an adjustable straight-back chair, the lower leg unsupported and the knee flexed to 90°. Force was measured with a strain gauge and recorded with a strain meter [18,19].

Explosive leg extensor power (both legs) was assessed with the Nottingham Power Rig. The subject, in a seated position with folded arms, gave a maximal push to a large foot pedal setting a flywheel in motion. The initial flywheel speed reflects the leg extensor power of the subject [20]. The measurement was repeated for at least five efforts, until no further improvements were seen.

Handgrip strength (both hands), which is associated with general muscle strength, was measured with a handgrip mechanical dynamometer [21]. The best of at least three attempts for each hand was recorded.

#### Bone mineral density (BMD)

BMD, measured by DEXA, was measured at the lumbar vertebrae (L1–L4, calculated from the total bone mineral content and the total area of the four lumbar vertebrae) and at the hip (neck, trochanter, and intertrochanteric region). DEXA scanner precision was carefully registered by means of a dedicated Hologic spine phantom. DEXA measurements were conducted at the Julius Center for Health Sciences and Primary Care, University Medical Center Utrecht, the Netherlands.

The following secondary parameters were used to determine the effect of the study medication:

#### Functional mobility, balance, endurance and body composition

Functional mobility was assessed quantitatively with the timed "get up & go" test [22]. The subject performed the test three times as fast as possible and the fastest time (recorded in seconds) was used for analysis.

Postural balance was assessed by means of an analysis of body sway and by the functional reach test. Body sway was assessed with both eyes open and both eyes closed [23]. In the Functional Reach the maximal push is recorded in cm [24].

Endurance was measured with the 6-minute walking test. (modified Cooper test) [25]. Body composition was measured by bio-electrical impedance analysis (BIA) and by DEXA. DEXA measurements were only performed at baseline and 24 months. The principle of BIA is that lean tissues, which mainly consist of electrolyte-containing water, readily conduct an applied current, whereas fat does not or only minimally. A current of 800 μA at a signal frequency of 50 Hz was generated by a bioelectrical impedance analyzer and was applied to the subject through electrodes that were placed on the wrist and the ankle on one side of the body [26]. Total body fat distribution and four sub-regions of leg fat distribution were determined from the DEXA scans [27,28].

#### Quality of life, well-being and mood/depression

Quality of life was measured with the Women's Health Questionnaire (WHQ) and the EQ-5D questionnaire [29,30]. The EuroQol (EQ-5D) questionnaire was developed by a European group as a standard non-disease-specific instrument for describing and valuing quality of life (EuroQol, Kind). It is a descriptive classification system consisting of five items, each with three levels. [31-33]

Mood/depression was measured with the Geriatric Depression Scale (GDS). This test is designed to assess for depression in older adults, independent of somatic symptoms [34,35]. It can also be used as indicator of severity of psychopathology.

Anxiety was measured with the Dutch Version of the State-Trait Anxiety Inventory (Self-Judgment Questionnaire = STAI-DY). The STAI was specifically designed to be self-administered. The test has a uniformly high level of internal consistency [36,37].

#### Cognitive function (15 Words test; Trails B test)

Cognitive function was assessed with the Groningen 15 Words test [38,39] and the Trails B test. The Groningen 15 Words test is an improved version of a test originally devised by Rey [40]. Briefly, 15 words are presented, one after another. Then the subject is asked to recall as many words as she can. This procedure is repeated 5 times, so that a learning curve can be plotted. The total number of words and the maximum number of words recalled over five trials are taken as dependent measures of learning and short-term memory. After a delay of 20 minutes (during which another test is administered), a recall trial is carried out without presentation of the list. The score at this delayed recall comprises the long-term memory score. The Trails B test measures speeded mental operations, attention, visual scanning, visual sequential abilities, and mental flexibility. The test is sensitive to aging effects [41,42].

### Compliance monitoring

Compliance with treatment was checked by means of a diary card and by counting the remaining tablets at each visit. Subjects who missed more than 6 days of trial medication in 4 weeks were excluded.

### Adverse event/Serious adverse event

The investigator decided whether an abnormal laboratory/vital sign was clinically relevant and should be entered on the adverse event form. If the AE met the definition of a serious adverse event (SAE), the procedure for SAEs was followed.

Serious adverse event was defined as any untoward medical occurrence that at any dose results in death, is life-threatening, requires in-patient hospitalization or prolongation of existing hospitalization, or results in persistent or significant disability/incapacity. It should be noted the term 'life-threatening' refers to an event in which the patient was at risk of death at the time of the event; it does not refer to an event that hypothetically might have caused death if it had been more severe.

Medical and scientific judgment was exercised in deciding whether major medical events that were not be immediately 'life-threatening' or result in death or hospitalization but which might jeopardize the patient or might require intervention to prevent one of the other outcomes listed in the definition above should be considered serious. All SAE's were reported to the Institutional Review Board of the University Medical Center of Utrecht, Utrecht, the Netherlands, to Organon NV, Oss the Netherlands and to Lilly Nederland B.V, Houten, the Netherlands.

### Power calculation

Changes in muscle strength and BMD are the primary endpoints to judge the efficacy of supplementation with both study drugs. In order to demonstrate a between-group difference in handgrip strength of at least 3.6% and a difference in BMD of at least 2.6%, a power calculation indicated 75 subjects per group completing the study. To allow for drop-out and protocol violations, it was calculated that 324 subjects would need to be recruited, 108 in each intervention arm. This number is based on conventional assumptions of alpha = 0.05 and beta = 0.2 and a drop-out of 30%. Mean and SD for handgrip strength were taken from our previous study on the effect of tibolone in postmenopausal women [13].

### Data analysis

#### Efficacy

Analysis will be done concerning tibolone versus placebo and raloxifene versus placebo. If both show a significant beneficial effect, the difference between tibolone and raloxifene will be analysed. However, this is not expected. After termination of the Tibolone-arm during our study, our concern is that the tibolone-placebo comparison will be underpowered. Analyses will be done with 3 months, 6 months en 12 months (n = 44) data to determine whether there is a treatment effect of tibolone. In previous studies with a comparable number of participants or fewer, a significant beneficial effect was reported on body composition after 1 year of treatment [43,44], so we might find an effect as well.

The efficacy analysis will be based on the intention to treat group (ITT), all subjects of the treated group who had at least one post baseline assessment of at least one of the primary outcome variables. In addition a per-protocol (PP) analysis will be performed, using data for all subjects from ITT group without any major protocol violation. Differences between the ITT and PP results will be examined.

#### Efficacy parameters

For all parameters, descriptive statistics will be calculated by treatment group and assessment. In addition, the change from baseline values will be calculated. If a Gaussian distribution can be assumed, each parameter will be analyzed by means of a multiple analysis of covariance model. This model will incorporate treatment group as a factor and the baseline value as a covariant. Scatter plots will be used to assess the adequacy of the analysis of covariance and to detect outliers. Results will be expressed as estimates of the treatment differences with 95%-confidence intervals and two-sided P-values.

#### Ethical and regulatory considerations

The study described in this protocol complies with the current revision of the Declaration of Helsinki, ICH guidelines for Good Clinical Practice and local regulatory requirements.

## Conclusion

This trial will analyse the differences in effect of tibolone and raloxifene on several endpoints. To the best of our knowledge this randomized controlled double-blind, placebo-controlled trial is the first to assess the main effect of tibolone and raloxifene on muscle strength and functional mobility next to bone mineral density in a female population older than 70 years of age. Research has never been conducted on the effect of raloxifene on muscle strength and functional mobility.

## List of abbreviations

HRT: hormone replacement therapy; SERMs: selective estrogen receptor modulators; ER: estrogen receptors, BMD: bone mineral density; MMSE: minimental state examination; DEXA: dual energy Xray absorptiometry; BIA: bio-electrical impedance analysis; WHQ: women's health questionnaire; HPA: habitual physical activity; STAI: state trait anxiety inventory; STAI-DY: self judgment questionnaire; GDS: geriatric depression scale; LIFT-study: Long term Intervention on Fractures with Tibolone; EQ: EuroQol, SAE: serious adverse event; AE: adverse event.

## Competing interests

The authors declare that they have no competing interests.

## Authors' contributions

HJJV designed the study, developed the research question, wrote the study protocol, obtained local ethics approval, obtained grant funding and implemented this study. HJJV, MMS and DEJ participated in its design and coordination. DEJ drafted the manuscript. HJJV, MMS, YTS and DEG helped to draft the manuscript. All authors have read and approved the final manuscript.
